# Unexpectedly high antibacterial ability of water in copper pot with tiny amount of plant leaves

**DOI:** 10.1016/j.wroa.2024.100238

**Published:** 2024-07-16

**Authors:** Min Zhang, Zhening Fang, Jun Wang, Rui Ding, Haiping Fang, Ruoyang Chen

**Affiliations:** a School of Physics, East China University of Science and Technology, Shanghai 200237, China; b Wenzhou Institute, University of Chinese Academy of Sciences, Zhejiang 325000, China; c Center for Transformative Science, ShanghaiTech University, Shanghai 201210, China

**Keywords:** Copper pot, Plant leaves, High antibacterial ability, Monovalent copper ions

## Abstract

Water disinfection by copper vessels has been prevalent over thousands of years. Unfortunately, people are still suffering from the bacterial pollution in drinking water. Here we show that, only through steeping with tiny amounts of common plant leaves, the room-temperature water in copper pots has unexpectedly high antibacterial ability. Remarkably, copper ions released from copper pots into water are in concentrations lower than the WHO safety threshold for drinking water, and have effective antibacterial ability when water contains specific leave components (polyphenols and/or lignin). Our computations show that the key to enhance antibacterial ability is the great increase in the proportion of Cu^+^ induced by aromatic rings in these leave components, which has been demonstrated by our experiments. The findings may disclose the mystery of copper vessels for water disinfection, and more importantly, provide effective antibacterial applications in industries and daily lives, by safely using copper ions together with biocompatible natural substances.

## Highlights

• The water in copper pots has unexpectedly high antibacterial ability with tiny amounts of common plant leaves.
• The proportion of monovalent copper ions is increased by aromatic rings in plant leaves.
• It promises effective antibacterial applications in industries and daily lives.

## Graphical abstract

**Figure:**
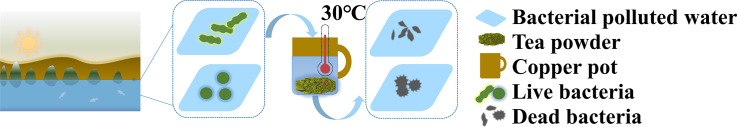

## Introduction

Water disinfection by using copper vessels has a long history over thousands of years, which can be dated back to the time of the Persian kings and later have been widely applied in various countries (Lemire et al., 2013; Moore et al., 1905). However, it still remains a severe sanitary problem of bacterial pollution in drinking water, which has been reported to cause more than 0.8 million deaths per year even in recent years and will be serious within next decades (Willyard, 2017).

From a scientific view, when copper vessels, e.g., copper pots, are holding water, copper ions can be released into water (Qian et al., 2023; Reuther et al., 2018). Recent studies have shown that copper ions in water had the antibacterial ability (Frei et al., 2023; Gross et al., 2019; Won et al., 2022), which was positively associated with their concentrations (Ermini and Voliani, 2021; Han et al., 2020; Song et al., 2021). However, the concentration of copper ions released from copper pots into water at room temperature is quite low, only ∼2 mg/L, which is insufficient for achieving the effective antibacterial activity on common bacteria in water, e.g., *E. coli* and *S. aureus* (Carlo et al., 2013).

On the other hand, the copper ion-based antibacterial materials have attracted increasing attention. However, the applications are limited by the safety threshold of 2 mg/L for copper ions in drinking water proposed by World Health Organization (WHO) (Jomova and Valko, 2011). To enhance the antibacterial ability of copper ions, various strategies have been proposed. It has been shown that the local concentration of copper ions increases by either directly mixing them with specific bio-extracts, e.g., antimicrobial peptides (AMPs) (Portelinha et al., 2021) or combining them with synthetic antibacterial agents, e.g., quinolone and pyridone (Mitscher, 2005; Psomas and Kessissoglou, 2013); however, the cytotoxicity of the sudden increase in the local concentration of copper ions and the instability of these composites have raised concerns (Chen et al., 2023; Fjell et al., 2012; Portelinha et al., 2021). Recently, the reduced graphene oxide (rGO) has been found to increase the proportion of monovalent copper ions and thus greatly enhance the antibacterial ability;(Tu et al., 2021) however, the toxicology of the rGO remains debated (Lu et al., 2019; Tu et al., 2013). Obviously, it is still in a great need of the safe strategy that can not only offer sufficiently strong antibacterial effects, but also keep copper ions in the WHO safety threshold together with biocompatible additives.

Here we show that, only through steeping with tiny amounts of common plant leaves, room-temperature water in copper pots has unexpectedly high antibacterial ability. We note that the concentration of copper ions released from copper pots into water (∼1.6 mg/L) is lower than the WHO safety threshold for drinking water and insufficient for achieving the effective antibacterial ability. Our experiments and computations show that some leave components rich with aromatic rings, e.g., polyphenols and lignin, can greatly increase the proportion of Cu^+^, and thus significantly enhance the antibacterial ability. We propose a facile and safe antibacterial technique for drinking water by safely using copper ions together with biocompatible natural substances. This antibacterial technique is not restricted in water disinfection, but has a wide range of applications in industries and daily lives, particularly in human body, e.g., mouthwash, hand sanitizer and antibacterial cosmetics, as well as sewage treatment for environmental protection.

## Results and discussion

### Unexpectedly high antibacterial ability

The fresh-picked, water-washed green tea leaves were oven-desiccated at 60 ℃, and then ground into tea powders using an agate mortar. Bacterial polluted water was prepared by diluting the *E. coli* or *S. aureus* with water to a cell density of 2 × 10^5^ colony forming units per milliliter. In a common copper pot, tea powders were steeped with bacterial polluted water in a concentration of only 4 mg/mL and kept under ambient conditions for 6 h (Tea water@Cu). We note that such a concentration is much lower than the typical concentration of the tea water of 10 mg/mL (Lambert and Elias, 2010). After incubation at 37 ℃ for 3 h, the agar plate counting test was performed to analyze the antibacterial efficacy against both bacteria (see details in Materials and methods).

**Fig. 1:**
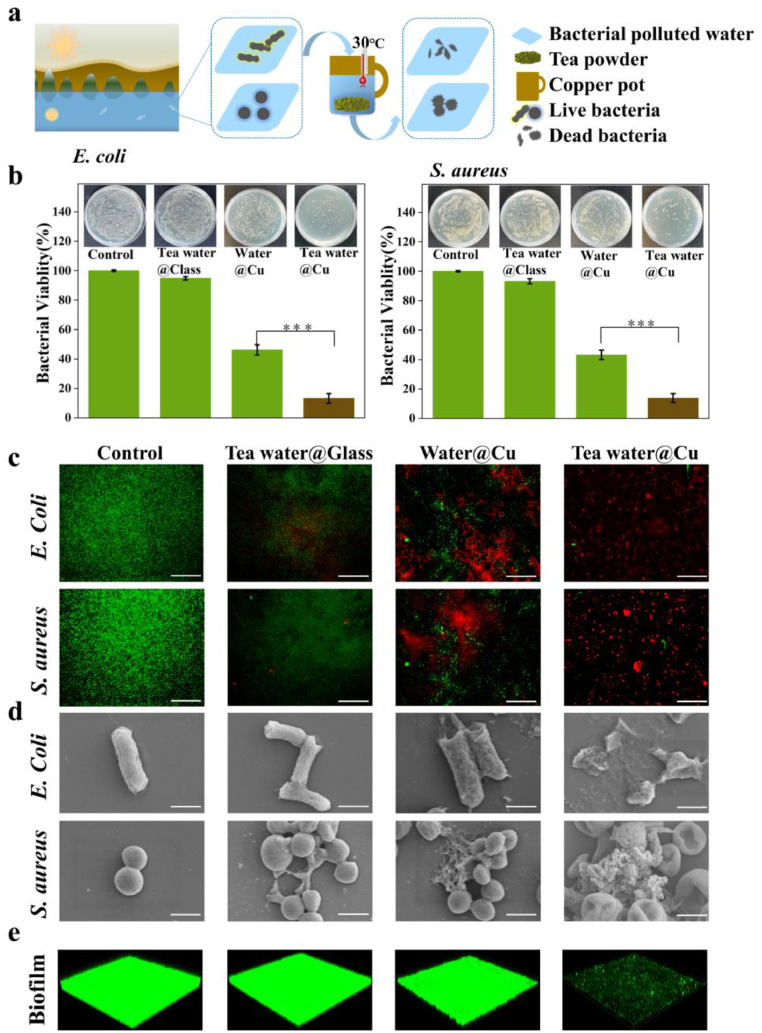
**Antibacterial effects of water in copper/glass containers with/without tea leaf.** (**a**) Schematics of antibacterial activities of tea water in a copper pot (Tea water@Cu): trace amounts of green tea powders were steeped with bacterial polluted water with a cell density of 2 × 10^5^ colony forming units per milliliter, in a concentration of only 4 mg/mL, under ambient conditions. (**b)** Bacterial viability and photographic images of colonies of *E. coli* and *S. aureus* in Tea water@Cu, tea water in a glassware (Tea water@Glass) and pure water in the copper pot (Water@Cu)*,* with bacteria in phosphate buffer saline (PBS) were set as control. (**c**) Live/dead viability assays of bacterial cells in different groups; living and dead cells were stained by green and red fluorescent dyes, respectively; scale bar: 50 µm. (**d**) Typical SEM images of dead bacterial cells in different groups; scale bar: 1 μm. (**e**) Three-dimensional (3D) reconstruction of biofilm of bacterial cells in Tea water@Glass, Water@Cu and Tea water@Cu by using a confocal laser scanning microscopy (CLSM); the biofilm of living cells was stained by green fluorescent dyes (1272 µm×1272 µm).

As shown in Fig. 1b, a very high antibacterial efficacy of 90±3 % against either *E. coli* or *S. aureus* was obtained, leaving only ∼10 % of living bacteria. We also performed experiments with tea water in a glassware (Tea water@Glass) and pure water in the copper pot (Water@Cu), respectively. The antibacterial efficacy of them is only 7 ± 1 % and 54±3 %, respectively, which is much lower than that of Tea water@Cu.

The typical fluorescent images of living and dead bacterial cells, stained by green and red fluorescent dyes, are shown in Fig. 1c. For Tea water@Cu, most bacterial cells were dead, leaving only few scattered living cells. However, for Tea water@Glass, most bacterial cells were alive, which was similar to the control group. For Water@Cu, we can see some dead cells together with many living cells. These are consistent with the antibacterial abilities we obtained.

The morphologies of dead bacterial cells were observed using scanning electron microscope (SEM) imaging. Most dead bacterial cells in Tea water@Cu were seriously damaged so that we could not clearly see the complete bacterial profile, as shown in Fig. 1d. However, most dead bacterial cells in Tea water@Glass and Water@Cu were still intact.

We also performed biofilm experiments on *S. aureus* (*E. coli* is difficult in biofilm formation) (Liu et al., 2023). The typical confocal laser scanning microscopy (CLSM) images in Fig. 1e revealed that Tea water@Cu had a much lower intensity of green fluorescence than others. The biofilm mass ratio was down to only 10 % in Tea water@Cu, much lower than 50 % in other groups (Supplementary Fig. S1). These findings demonstrate that Tea water@Cu not only kills bacterial cells, but also has significant biofilm inhibition effects. (Kim et al., 2017; Xiu et al., 2022). Furthermore, we have performed the toxicity examination on Tea water@Cu against mammalian cells by using a CCK-8 assay. Our experimental results in Fig. S2 clearly show that the cell viability of Tea water@Cu is above 85 %, indicating the high biocompatibility of Tea water@Cu.

### Main contributors to high antibacterial ability

To figure out main contributors to high antibacterial ability of Tea water@Cu, we first measured the concentration of copper ions by using the inductively coupled plasma mass spectrometry (ICP-MS). Only 1.6 ± 0.2 mg/L of copper ions were released from the copper pot into both Water@Cu and Tea water@Cu. Our experiments on water with 1.6 mg/L of copper ions show almost the same antibacterial ability of 54±3 % as the case of Water@Cu, which leaves ∼46 % of living bacteria, indicating that only Water@Cu is insufficient for achieving the effective antibacterial activity.

**Fig. 2:**
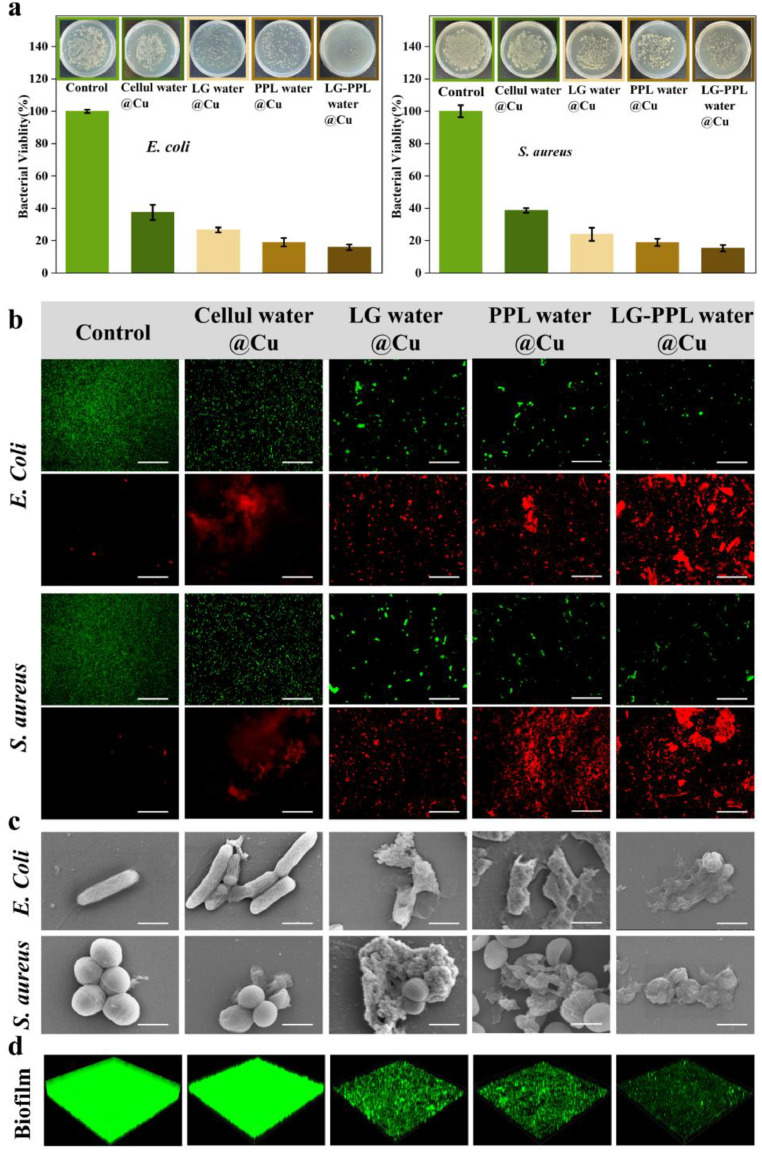
**Antibacterial effects of water with copper ions when steeping with different components of tea leaf.** (**a**) Bacterial viability and photographic images of colonies of *E. coli* and *S. aureus* in water with copper ions when separately steeping with the single component of lignin (LG water@Cu), polyphenols (PPL water@Cu), cellulose (Cellul water@Cu), and the coexistence of lignin and polyphenols (LG-PPL water@Cu). (**b**) Live/dead viability assays, with living and dead bacterial cells stained by green and red fluorescent dyes, respectively, scale bar: 50 µm. (**c**) Typical SEM images of dead bacteria, scale bar: 1 μm. (**d**) Three-dimensional (3D) reconstruction of biofilm of bacterial cells in LG water@Cu, PPL water@Cu, Cellul water@Cu, and LG-PPL water@Cu by using a confocal laser scanning microscopy (CLSM); the biofilm of living cells was stained by green fluorescent dyes (1272 µm×1272 µm).

The green tea leaf in our experiments contains 15wt % of polyphenols (PPL), 36wt % of lignin (LG) and 7wt % of cellulose (Cellul). Based on their contents, we separately steeped only one of them into water with 1.6 mg/L of copper ions, which is same as the concentration of copper ions released in Tea water@Cu, for achieving the concentrations of PPL, LG and Cellul at 0.6 mg/mL, 1.44 mg/mL and 0.28 mg/mL, and named as PPL water@Cu, LG water@Cu and Cellul water@Cu, respectively. As shown in Fig. 2a, they exhibited the antibacterial efficacy of 84±2 %, 80±3 %, and 55±2 %, respectively. The antibacterial efficacy of PPL water@Cu and LG water@Cu was much higher than that of Water@Cu (54±3 %), but still much lower than that of Tea water@Cu of 90±1 %. However, the antibacterial efficacy of Cellul water@Cu was as low as that of Water@Cu. We have also performed experiments on water only with PPL, LG or Cellul. The antibacterial efficacy of these individual components in pure water was negligibly low, with only 3 ± 2 % (Fig. S3). These results demonstrate that, in the presence of copper ions, LG and PPL are two important leaf components for high antibacterial efficacy.

Considering that the tea leaf contains both LG and PPL, we further performed experiments on the coexistence of LG and PPL in water with 1.6 mg/L of copper ions, namely LG-PPL water@Cu. As expected, the antibacterial efficiency of LG-PPL water@Cu against both *E. coli* and *S. aureus* significantly increases to 88±2 % (Fig. 2a), approaching to that of Tea water@Cu. We think that other low-content components, e.g., alkaloids and indoles, in tea leaf, might further enhance the antibacterial ability (Suganya et al., 2022).

We have further found that, similar to Tea water@Cu, most bacterial cells in LG-PPL water@Cu, LG water@Cu and PPL water@Cu were dead with seriously physical damage (Fig. 2b and c). They also have significant effects on biofilm inhibition, similar to Tea water@Cu (F Fig. 2d and Fig. S4).

By increasing the concentration of LG and/or PPL, the antibacterial efficiency of water with 1.6 mg/L of copper ions can be further enhanced (Fig. S5). Our experiment shows that the antibacterial efficiency is over 90 % for both PPL water@Cu with 1.5 mg/mL of PPL and LG water@Cu with 7.2 mg/mL of LG. It should be noted that concentrations of copper ions, PPL and LG are all lower than the safety threshold (Yang and Zhang, 2019). These results show that water with copper ions below the WHO safety threshold can have effective antibacterial ability by safely adding tiny amounts of biocompatible PPL and/or LG, and it can be developed as a safe, facile antibacterial technique for industrial applications particularly in human body and environmental protection.

### Mechanism of high antibacterial ability

**Fig. 3:**
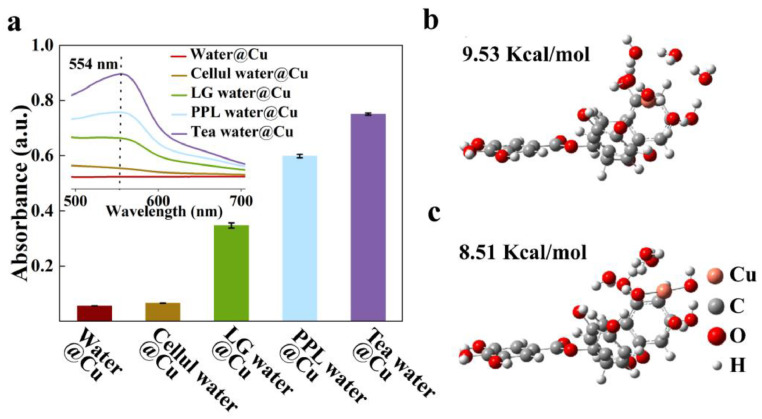
**Proportion analysis on monovalent copper ions (Cu^+^).** (**a**) UV–vis absorbance of Cu^+^ at 554 nm for Water@Cu (red), Cellul water@Cu (brown), LG water@Cu (green), PPL water@Cu (blue) and Tea water@Cu (purple) after being treated with bicinchoninic acid (BCA); inset shows their UV–vis spectra in the range of 500 nm to 700 nm. (**b**) Optimized structure for Cu^+^ wih polyphenol molecule. (**c**) Optimized structure for Cu^2+^ with polyphenol molecule.

It has been reported that monovalent copper ions (Cu^+^) had more significant antibacterial effects than divalent copper ions (Cu^2+^) when they are in the same concentration (Gross et al., 2019; Tu et al., 2021). We analyzed the proportion of Cu^+^ in Tea water@Cu and Water@Cu by using the bicinchoninic acid (BCA) method (details in Methods). As shown in Fig. 3a, Tea water@Cu has a broad UV adsorption peak of Cu^+^ at 554 nm with a very high intensity of 0.72±0.05. By contrast, Water@Cu has a very low intensity of only 0.02±0.01. These indicate that Tea water@Cu has a much higher proportion of Cu^+^. We also performed the X-ray photoelectron spectroscopy (XPS) analysis. Since the XPS detection requires dried samples, we freeze-dried Tea water@Cu. It exhibits a peak at 931.50 eV (Fig. S6), which is the result of the overlapping of characteristic peaks of Cu^+^ at 934.04 eV and Cu^2+^ at 930.92 eV. The XPS spectral analysis indicates that the ratio of Cu^+^ and Cu^2+^ is ∼1:2.

We then analyzed the proportion of Cu^+^ in LG water@Cu and PPL water@Cu. Fig. 3a clearly show that they all have the UV adsorption peak of Cu^+^ at 554 nm with intensities of 0.35±0.01 and 0.50±0.05, respectively, indicating high proportion of Cu^+^ in LG water@Cu and PPL water@Cu. Here we only performed XPS analysis on the freeze-dried LG water@Cu, since PPL dissolved well in water, which was inappropriate for XPS analysis. We found that the ratio of Cu^+^ and Cu^2+^ in LG water@Cu was ∼1:2 Fig. S6). These results are consistent with their antibacterial ability in Fig. 2a.

We next performed the density functional theory (DFT) calculation for the increase in the proportion of Cu^+^. Here we only show the computational results of polyphenol, since the lignin molecule is too big to obtain a reliable result, using quantum mechanism computation. As shown in Fig. 3b and c, a cluster of one Cu cation and six water molecules was set near the aromatic rings of the polyphenol. The B3lyp was selected as the calculation method. The lanl2dz was chosen as basis for the Cu cation, and 6–31G* was for other atoms. To reproduce the natural states of the cations, 1 (2) water molecule was replaced with hydroxide ions for the calculation of Cu^+^(Cu^2+^). By choosing a large number of initial candidate geometries, the optimized structure was determined. The calculated adsorption energy for the Cu^+^(Cu^2+^) system was 9.53 (8.51) kcal/mol, which tells that the polyphenol molecule indeed helps increasing the proportion of Cu^+^ in the water. All computation results are consistent with our experimental results, indicating that the enhanced antibacterial activity is correlated to the increased proportion of Cu^+^ induced by polyphenols in tea leaf when they are steeping into water with copper ions.

### Universality of high antibacterial effects

**Fig. 4:**
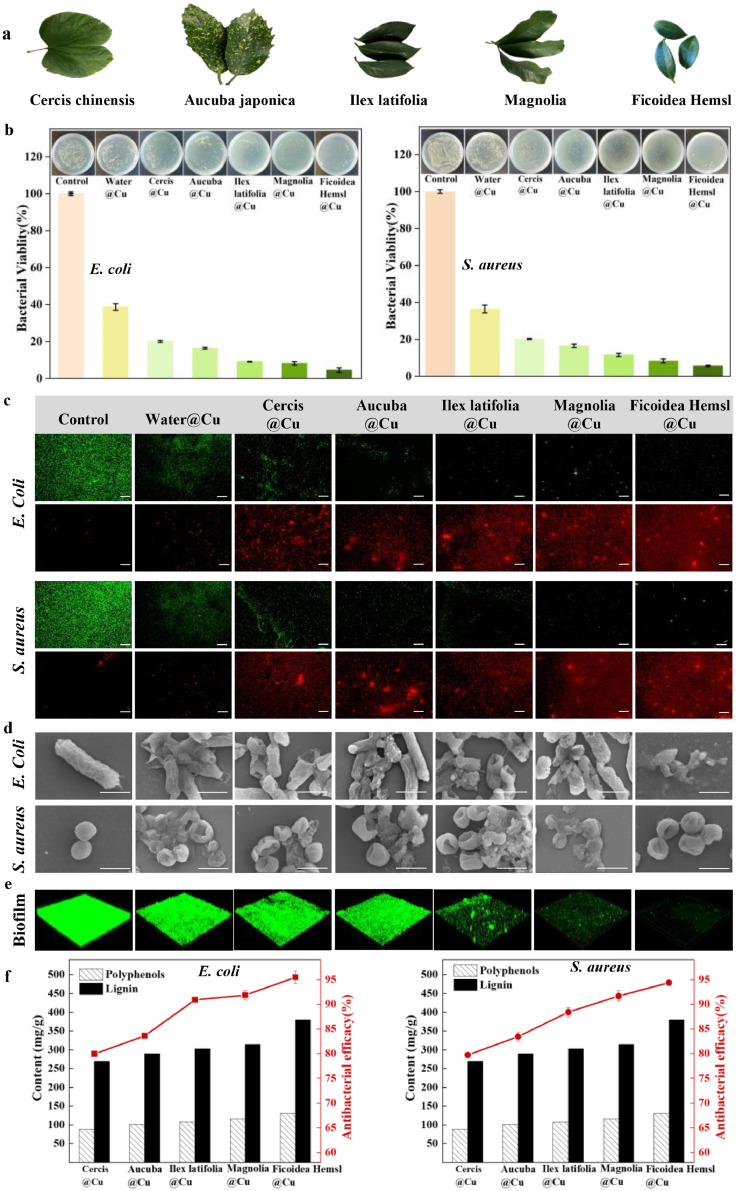
**Antibacterial effects of water with copper ions when steeping with plant leaves rich in polyphenols and lignin.** (**a**) Images of plant leaves of Cercis chinensis, Aucuba japonica, Ilex latifolia, Magnolia, Cercis chinensis and Ficoidea Hemsl. (**b**) Bacterial viability and photographic images of colonies of *E. coli* and *S. aureus* in water with copper ions when steeping with plant leaves. (**c**) Living and dead bacterial cells stained by green and red fluorescent dyes, respectively, scale bar: 50 µm. (**d**) Typical SEM images of dead bacteria, scale bar: 1 μm. (**e**) Three-dimensional (3D) reconstruction of biofilm of bacterial cells in water with copper ions when steeping with plant leaves by using a confocal laser scanning microscopy (CLSM); the biofilm of living cells was stained by green fluorescent dyes (1272 µm×1272 µm). (**f**) The content of polyphenols (white bar) and lignin (black bar) in leaves, and the antibacterial efficacy (red line) of water with copper ions when steeping with these leaves in a concentration of 4 mg/mL.

The high antibacterial effects are universal in various fresh-picked plant leaves that are rich in polyphenols and lignin. We show examples of leaves of Cercis chinensis, Aucuba japonica, Ilex latifolia, Magnolia, and Ficoidea Hemsl (Fig. 4a). As shown in Figs. 4b-d, the antibacterial efficacies of the water in copper pot with these leaves in a concentration of 4 mg/mL(similar to Tea water@Cu) are 84±1 %, 87±2 %, 89±1 %, 81±1 % and 90±1 %, respectively. We also observed that the plant leaves in copper pot were effective at preventing the formation of bacterial biofilm in *S. aureus*, only 10 %∼20 % bacterial viability was observed in biofilm (Fig. 4e). By contrast, the plant leaves steeped into pure water have a very low antibacterial efficacy (< 10 %), showing negligible antibacterial ability (Fig. S7). As shown in Fig. 4f, there is a positive correlation between the antibacterial ability and the contents of LG and PPL in these leaves.

Furthermore, we gave a counterexample by steeping holly twigs, which contain much less polyphenols (77 mg/g) and lignin (225 mg/g) than plant leaves (Fig. S8 and Fig. S9), into copper pot water. In contrast, the antibacterial efficacy is 65 %, only raises ∼5 % in the case of holly twigs in pure water. Our findings demonstrate that the high antibacterial effect is universal in plant leaves that are rich in polyphenols and lignin, when they are steeped into water in a copper container.

## Conclusions

In summary, we have found that room-temperature water with trace amounts of copper ions (1.6 mg/L), which even would be easily achieved by holding water in regular copper pots, possessed unexpectedly high antibacterial ability, only through steeping with tiny amounts of common plant leaves. The concentration of copper ions is lower than the WHO safety threshold (2.0 mg/L) for drinking water and insufficient for acheiving effective antibacterial effects. Our experiments and computations have shown that polyphenols and lignin in plant leaves greatly increased the proportion of Cu^+^, and thus significantly enhanced the antibacterial ability. Remarkably, tea leaf (4 g/L), polyphenols (0.6 g/L) and lignin (1.44 g/L) are all in safe concentration thresholds, but have very weak antibacterial effects when they are in pure water without copper ions. We have further demonstrated that the high antibacterial effect was universal in plant leaves that were rich in polyphenols and lignin, when they were steeped into water in the copper pot.

Our research may disclose the mystery of the long-term popularity of water storage by using copper vessels, i.e., high antibacterial ability could be obtained when plant leaves were accidentally steeped into water in copper vessels. It also offers new insights into the transformation in valence states of metal cations induced by aromatic rings, which is the previously unrecognized but ubiquitous phenomenon in nature.

We note that copper vessels here are only one of common examples to obtain low concentration of copper ions; essentially we develop an effective antibacterial technique, by safely using copper ions together with biocompatible natural substances, e.g., polyphenols and/or lignin. This technique should have a wide range of industrial applications particularly in human body, e.g., mouthwash, hand sanitizer, and antibacterial cosmetics, as well as sewage treatment for environmental protection. It also can be used for emergency cases requiring safe drinking water, e.g., outdoor adventure tourism and outdoor rescue, in which water is from the underground with a high level of bacteria, on one hand, and antibacterial conditions are very limited, on the other hand(He et al., 2023; Venkatesha et al., 2020). People can easily achieve the safe drinking water through steeping tiny amounts of plant leaves into water in copper vessels.

Overall, our findings may present an understanding of the long history of using copper vessels for water disinfection, and more importantly, represent a big step in developing a facile and safe antibacterial technique, which is suitable for emergency cases requiring safe drinking water and industrial applications.

## Materials and methods

### Materials

Copper (II) chloride (CuCl_2_) and Copper (I) chloride (CuCl) were supplied by Aladdin Bio-Chem Technology (Shanghai, China). Green tea leaves were sourced from Guangxi, China and other leaves were obtained from Wenzhou Institute, University of Chinese Academy of Sciences. Lignin Content Assay Kit and Lignin Content Assay Kit were purchased from Solarbio (Beijing, China). LB Agar and Trypticase Soy Broth (TSB) were provided by Qingdao Hope Bio-Technology (QingDao, China). Phosphate buffer saline (PBS) was obtained from Beijing Solarbio Science & Technology (Beijing, China). Calcein-AM/PI Double Stain Kit and SYTO9/PI Double Stain Kit were from Shanghai Maokang Biotechnology (Shanghai, China). Dulbecco's Modified Eagle's Medium (DMEM) were from Thermo Fisher Scientific (Shanghai, China) and Cell Counting Kit-8 (CCK-8) was purchased from Dojindo (Shanghai, China). *S. aureus* (ATCC 43300) was purchased from Shihai Luwei Technology (Shanghai, China) and *E. coli* (ATCC 25922) was from Shanghai Bioresource Collection Center (Shanghai, China). Fibroblast cells (L929) were obtained from Procell Life Science & Technology (Wuhan, China)**.**

### Preparation of tea water@Cu powder

Fresh leaves were chosen to remove any dirt or debris and the stems were removed. 0.04 g of leaves were dried and ground into powder and then dispersed into 10 mL of ultrapure water (18.25 MΩ·cm) produced from a purification system (Ulupure, Sichuan of China) in the copper pot. The mixture was sealed and stored still at room condition for 12 h and then vacuum filtered onto an ultrafilter membrane (50 mm diameter, 0.22 μm pore size). The Tea water@ Cu sediments were dried and store the powder in an airtight container for experimental measurements.

### *In vitro* cytotoxicity assay

L929 fibroblast cells were inoculated into 96-well plates (1×10^3^ cells per well) containing different nutrient solutions, i.e., saline (control), Tea water@Class (4 mg/mL), Water@Cu (Cu: 1.6 mg/L) and Tea water@Cu (Cu: 1.6 mg/L, Tea: 4 mg/mL). After culturing for 48 h, the nutrient solutions were removed and the cells were gently washed with phosphate buffer saline (PBS) for three times. Then, the cells were incubated with 100 μL of fresh culture medium containing 10 % CCK-8 (v/v) for 2 h. The optical density (OD) of each well was analyzed at 450 nm on a Thermo Scientific Varioskan LUX Multimode Microplate Reader. The cell viability was expressed as a relative percentage compared to the untreated group. The living state of bacteria was analyzed by using a ZEISS Axio Vert.A1 confocal laser scanning microscopy imaging.

### Antimicrobial efficacy of tea water@ Cu

Bactericidal efficacy tests: 0.4 g of leaves powder dispersed into 10 mL of copper pot water that contained bacterial suspensions (2 × 10^5^ colony forming units (CFU)/mL *E. coli* or *S. aureus)* at 37 ℃ for 6 h. On one hand, 100 µL of bacterial suspensions were serially incubated in 96-well plates at 37 °C (*E. coli* in 2 ×LB or *S. aureus* in 2 ×TSB) for 24 h and OD600 measured (Thermo Scientific Varioskan LUX Multimode Microplate Reader) for bacterial density estimation. On the other hand, the bacterial cultures were serially diluted with sterile PBS were plated on TSB agar and 100 µL of each diluted sample was spread on the tryptic soy agar plates to observe the number of colonies. The bacterial viability assay on each group was repeated for at least three times. The bacterial viability and its standard deviation were calculated based on these repeating experiments.

Live/dead bacterial viability assays: The samples were prepared by a similar procedure as mentioned above. 20 μL LIVE/DEAD BacLight Bacterial Viability Kit was added to the incubation solution and observed by a ZEISS Axio Vert.A1 fluorescent microscopy imaging system after incubation for 15 min. Live bacteria with intact membranes display green fluorescence and bacteria with disrupted membranes fluoresced red.

Biofilms inhibition tests: Bacterial suspensions (100 µL) were separately mixed with saline, Tea water@Glass, Water@Cu and Tea water@Cu, and then incubated in 96-well plates at 37 °C (S. aureus in 2 ×TSB). After incubation for 48 h, the liquid nutrient medium and free-floating bacteria were removed by inverting the plates, and the wells were washed vigorously with doubly distilled water (DDW) for at least three times. Then, 100 µL of 0.05 wt % crystal violet was added to each well and incubated at 37 ℃ for 15 min, followed by another three runs of DDW wash to remove the unbound dye. The biofilm formation was quantified by measuring the difference in absorbance at 550 nm using a Thermo Scientific Varioskan LUX Multimode Microplate Reader after adding 200 µL of 30 % acetic acid to each well for 15 min to dissolve the dye bounded onto cells. To visualize the activity of the bacterial biofilms, live/dead staining was performed. The biofilms treated by different methods were thoroughly washed and stained with the LIVE/DEAD Baclight Bacterial Viability Kit, and finally, visualized by the Nikon A1 confocal laser scanning microscopy imaging system.

### Morphological analyses of bacteria and biofilms by SEM images

Bacteria and biofilms after incubations with copper-leaves were dropped onto silicon wafers and fixed with 2.5 % glutaraldehyde solution for 3 h. Samples were dehydrated with a graded series of ethanol solutions (30 %, 50 %, 70 %, 90 % and 100 %, 15 min each step). The morphology of the bacteria was observed with a Hitachi Su 8010 instrument at 5.0 kV after being dried under a stream of nitrogen gas and coated with an ultrathin gold coating by sputtering.

### Characterization methods

The inductively coupled plasma mass spectrometry (ICP-MS, Agilent 7850) with an SPS-4 autosampler (Agilent) was used to further analyze the concentrations of Cu in copper pot water. The sample were carefully diluted with the 2 % HNO_3_, prior to the ICP-MS analysis. 2 % HNO_3_ solution was used as the control of the analytical method. XPS measurements were performed with a spectrometer from ESCALAB Xi+ (Thermo Fischer). The monochromatized Al Kα X-ray source (*E* = 1486.6 eV) was operated at 12.5 kV and the filament current is 16 mA. The full spectrum of Passing Energy was 100 eV and narrow spectrum was 20 eV. The Cu^+^ ion was detected by using the bicinchoninic acid kit, i.e., the analyte solution was mixed with the bicinchoninic acid for 60 min at 37 °C, and then the UV–Vis adsorption was detected by using a CARY5000 spectrophotometer (Agilent) (Clemente et al., 2020). The detection mechanism is associated to the formation of chelate complex between one Cu^+^ ion and two bicinchoninic acid molecules; such a chelate complex can dissolve in water to produce the solution in purple with an obvious absorbance at 554 nm, and its intensity increases linearly with the concentration of complex.

### Setup for the DFT calculations

The calculation method for DFT was chosen as B3lyp for both optimization and energy calculations, since it is enough to describe the leading factors that lead to the adsorption. The basis for the Cu^+^/Cu^2+^ cation was chosen as lanl2dz, while 6–31G* was chosen for other atoms. To reproduce the natural states of the cations, 1 (2) water molecule was replaced with hydroxide ions for the calculation of Cu^+^ (Cu^2+^). By choosing a large number of initial candidate geometries, the best structure was determined and optimization was done.

### Analysis of components content in leaves

The basic active components of lignin, polyphenols and cellulose in leaves were measured by coupling the applications of each component content assay kit with the UV–Vis spectrum analysis. The detailed processes were according to the manufacturer's instructions.

## CRediT authorship contribution statement

**Min Zhang:** Writing – original draft, Data curation. **Zhening Fang:** Conceptualization. **Jun Wang:** Formal analysis. **Rui Ding:** Writing – original draft. **Haiping Fang:** Writing – review & editing. **Ruoyang Chen:** Writing – review & editing, Investigation.

## Declaration of competing interest

The authors declare that they have no known competing financial interests or personal relationships that could have appeared to influence the work reported in this paper.

## Data Availability

Data will be made available on request.

## Appendix Supplementary materials

Supplementary materials. The following files are available free of charge. Biofilms inhibitory potency of Tea water@Cu Toxicity of Tea water@Cu Viability of bacteria with LG, PPL, Cellul and the LG-PPL. Biofilms inhibitory potency of water with copper ions when steeping with different components of tea leaf. Antibacterial effects of Tea water@Cu, PPL water@Cu and LG water @Cu with different concentration. Valent analysis of Tea water @Cu, LG water@Cu and Cellul water@Cu. Antibacterial activities of the leaves. The content of lignin and polyphenols in leaves and twigs. Antibacterial activities of the Twigs water@Cu. Image, application 1
