# Supplementary material for: Unexpectedly high antibacterial ability of water in copper pot with tiny amount of plant leaves
Source: Water Res X. 2024 Jul 16;24:100238. doi: 10.1016/j.wroa.2024.100238 (PMC11327398; doi:10.1016/j.wroa.2024.100238)
Supplement: Supplementary file 1 — Supplementary materials. The following files are available free of charge. Biofilms inhibitory potency of Tea water@Cu Toxicity of Tea water@Cu Viability of bacteria with LG, PPL, Cellul and the LG-PPL. Biofilms inhibitory potency of water with copper ions when steeping with different components of tea leaf. Antibacterial effects of Tea water@Cu, PPL water@Cu and LG water @Cu with different concentration. Valent analysis of Tea water @Cu, LG water@Cu and Cellul water@Cu. Antibacterial activities of the leaves. The content of lignin and polyphenols in leaves and twigs. Antibacterial activities of the Twigs water@Cu. [file mmc1.docx]

**Supplementary Information**

**Unexpectedly high antibacterial ability of water in copper pot**

**with tiny amount of plant leaves**

**Authors:** Min Zhang^1,2†^, Zhening Fang^3†^, Jun Wang^1,2^, Rui Ding^1,2^, Haiping Fang^1,2^, Ruoyang Chen^1,2*^,

**Affiliations:**

^1^School of Physics, East China University of Science and Technology; Shanghai 200237, China

^2^Wenzhou Institute, University of Chinese Academy of Sciences; Zhejiang 325000, China

^3^Center for Transformative Science, ShanghaiTech University, Shanghai 201210, China

†These authors contributed equally to this work.

*Corresponding author. Email: [Ruoyang.chen@ecust.edu.cn](mailto:Ruoyang.chen@ecust.edu.cn)

**Table of Contents**

[Supplementary Information Section 1 Biofilms inhibitory potency of Tea water@Cu 3](#_Toc171674970)

[Supplementary Information Section 2 Toxicity of Tea water@Cu 4](#_Toc171674971)

[Supplementary Information Section 3 Viability of bacteria with LG, PPL, Cellul and the LG-PPL. 5](#_Toc171674972)

[Supplementary Information Section 4 Biofilms inhibitory potency of water with copper ions when steeping with different components of tea leaf. 6](#_Toc171674973)

[Supplementary Information Section 5 Antibacterial effects of Tea water@Cu, PPL water@Cu and LG water @Cu with different concentration. 7](#_Toc171674974)

[Supplementary Information Section 6 Valent analysis of Tea water @Cu, LG water@Cu and Cellul water@Cu. 8](#_Toc171674975)

[Supplementary Information Section 7 Antibacterial activities of the leaves 9](#_Toc171674976)

[Supplementary Information Section 8 The content of lignin and polyphenols in leaves and twigs 10](#_Toc171674977)

[Supplementary Information Section 9 Antibacterial activities of the Twigs water@Cu. 11](#_Toc171674978)

**Supplementary Information Section 1 Biofilms inhibitory** **potency of Tea water@Cu**


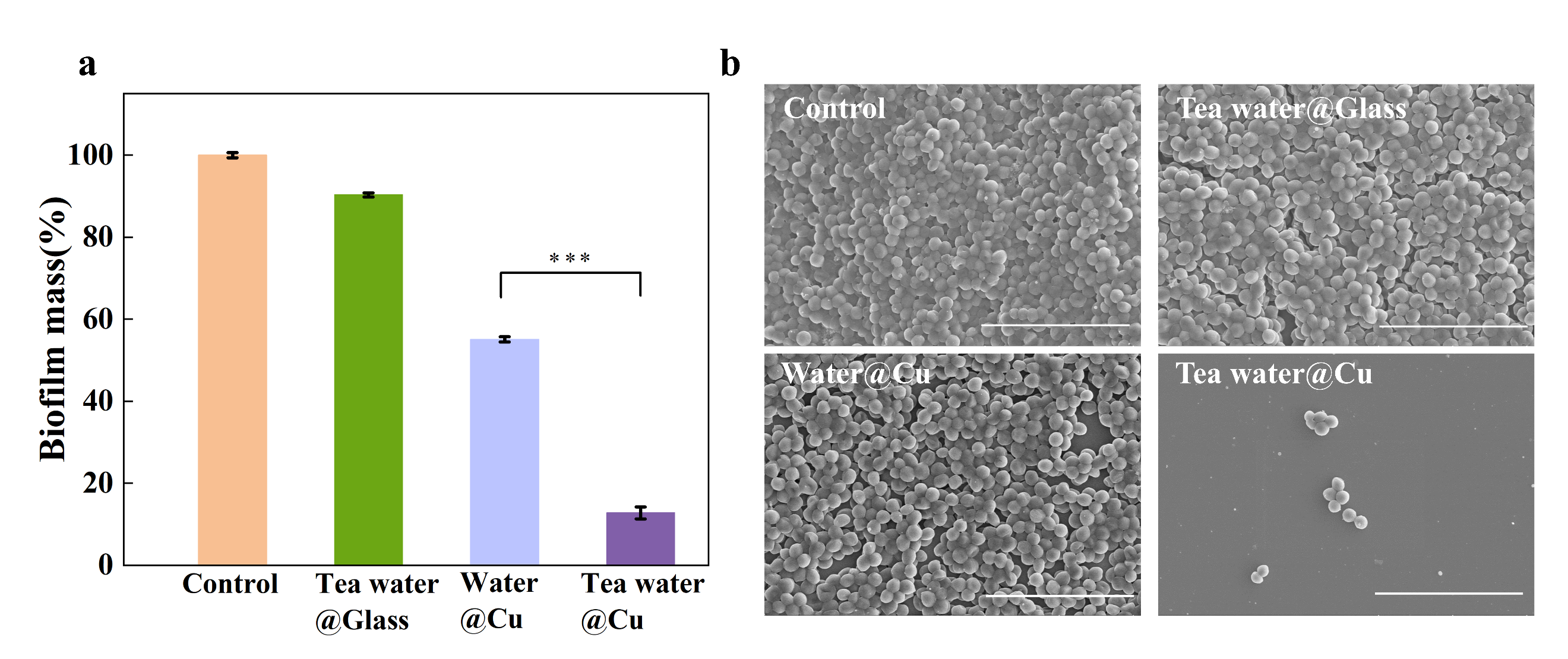


**Fig. S1.** (**a**) Quantitative analysis of *S. aureus* biofilms inhibitory potency after being treated by PBS, Tea water@Glass, Water@Cu and Tea water@Cu through measuring the optical density at 550 nm after crystal violet treatment. (**b**) SEM images of *S. aureus* biofilms after being treated by various methods (scale bar: 10 μm).

**Supplementary Information Section 2** **Toxicity of Tea water@Cu**


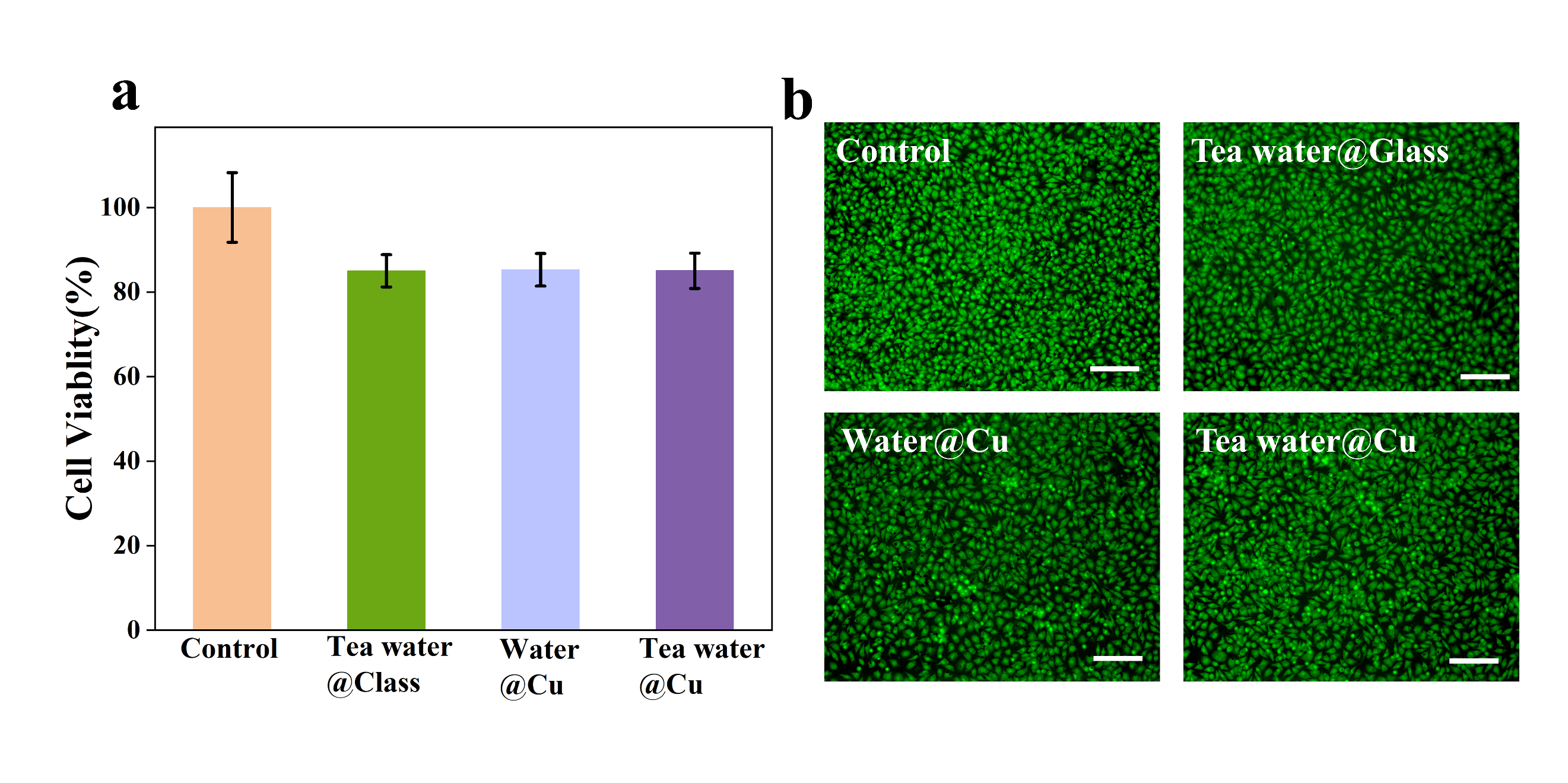


**Fig. S2.** (**a**) Cell viability of when treated with saline, Tea water@Class (4 mg/mL), Water@Cu(1.6 mg/L) and Tea water@Cu(Cu: 1.6 mg/L;Tea: 4 mg/mL). (**b**) Fluorescence images of L929 fibroblast cells when treated with saline, Tea water@Glass, Water@Cu and Tea water@Cu (Viable cells are green fluorescent and dead cells are green fluorescent, scale bar=20 μm).

**Supplementary Information Section 3 Viability of bacteria with LG, PPL, Cellul and the LG-PPL.**


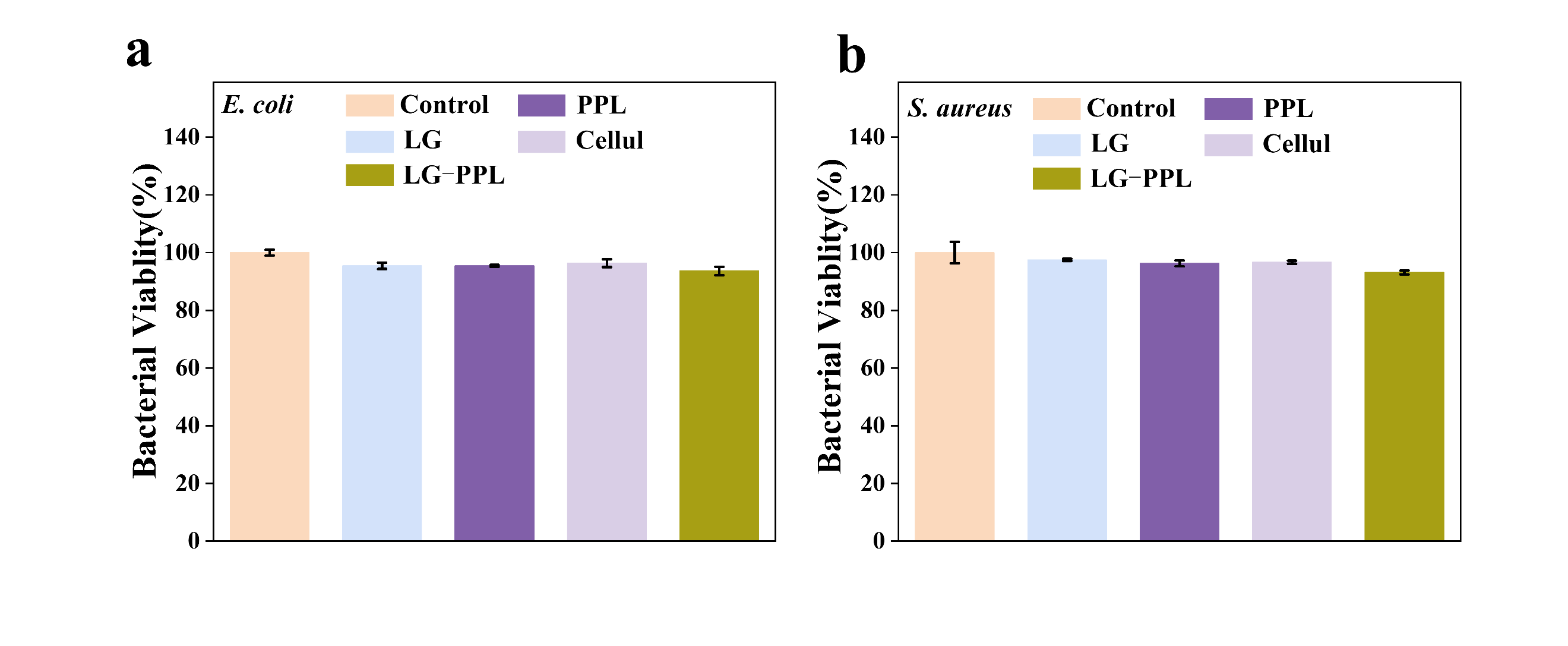


**Fig. S3.** Viability of *E. coli* (**a**) and *S. aureus* (**b**) with LG, PPL, Cellul and the LG-PPL, respectively.

**Supplementary Information Section 4** **Biofilms inhibitory potency of water with copper ions when steeping with different components of tea leaf.**


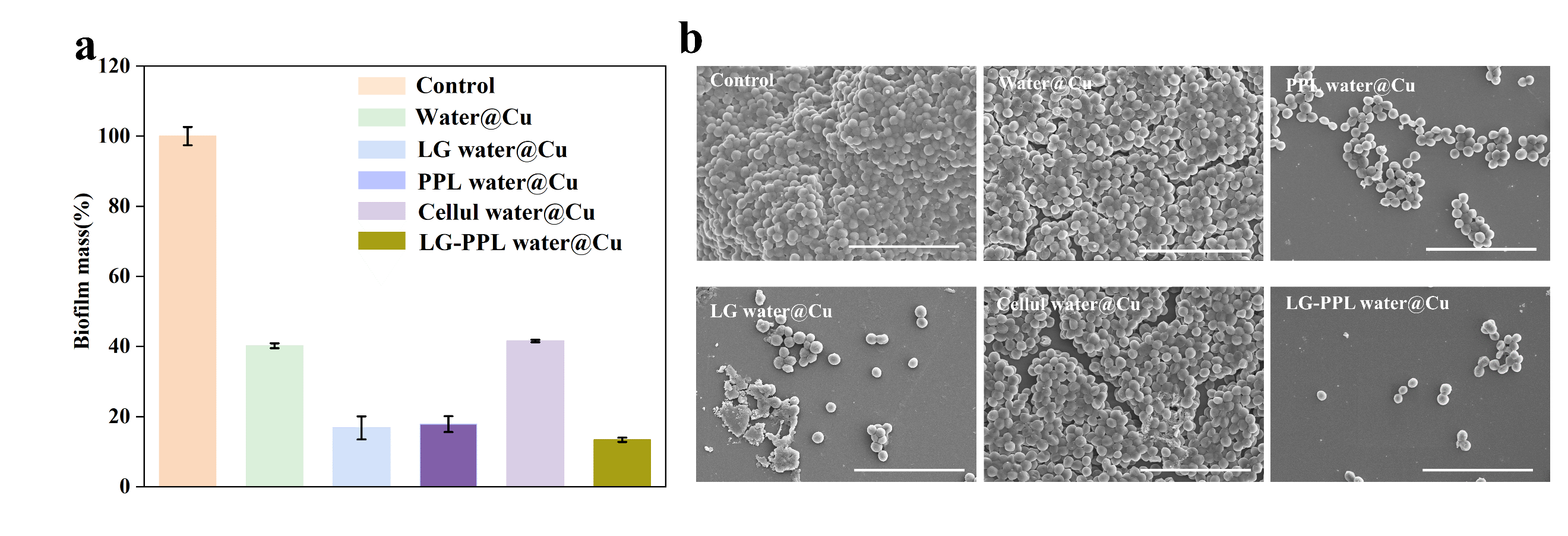


**Fig. S4.** (**a**) Quantitative analysis of *S. aureus* biofilms inhibitory potency after being treated by different methods through measuring the optical density at 550 nm after crystal violet treatment. (**b**) SEM images of *S. aureus* biofilms after being treated by various methods (scale bar: 10 μm).

**Supplementary Information Section 5 Antibacterial effects of Tea water@Cu, PPL water@Cu and LG water @Cu with different concentration.**


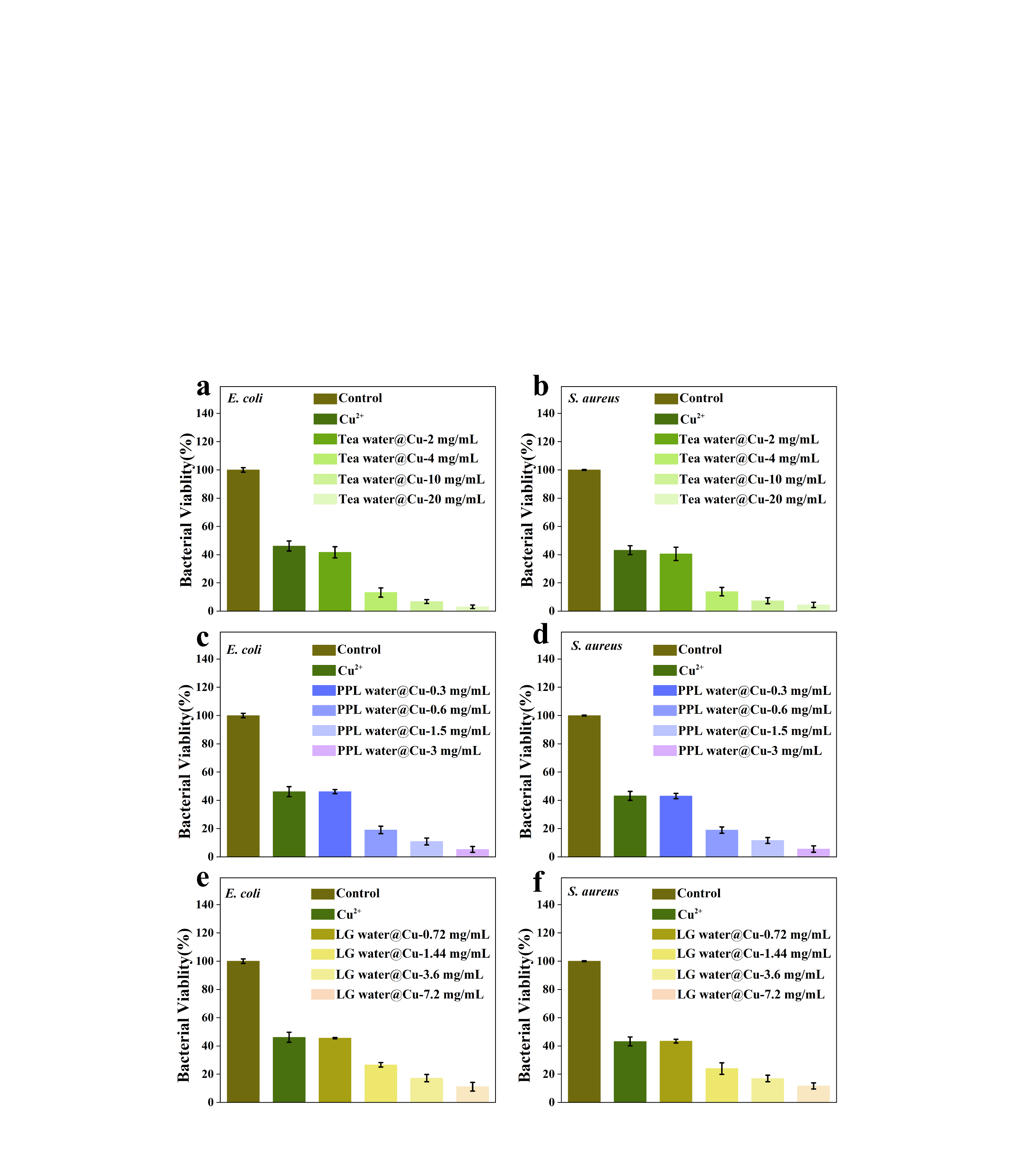


**Fig. S5.** Bacteria viability of *E. coli* and *S. aureus* with Tea water@Cu, PPL water@Cu and LG water @Cu with different concentration, respectively.

**Supplementary Information Section 6 Valent analysis of Tea water @Cu, LG water@Cu and Cellul water@Cu.**


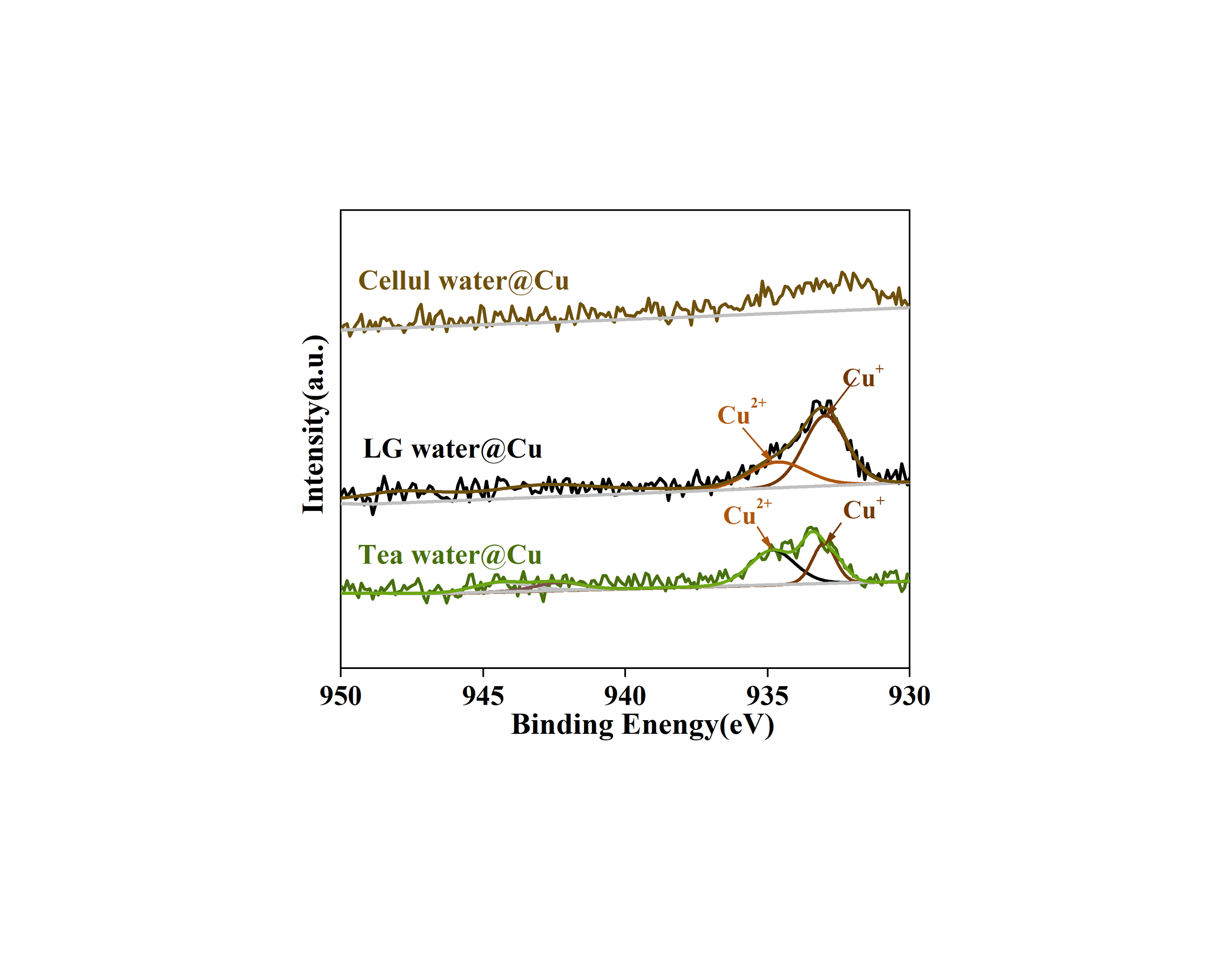


**Fig. S6.** XPS spectra of the Cu 2p peak of the Tea water @Cu, LG water@Cu and Cellul water@Cu. The peaks at ≈934.04 eV and ≈930.92 eV corresponding to the Cu^2+^ and Cu^+^ characteristics, respectively.

**Supplementary Information Section 7 Antibacterial activities of the leaves**


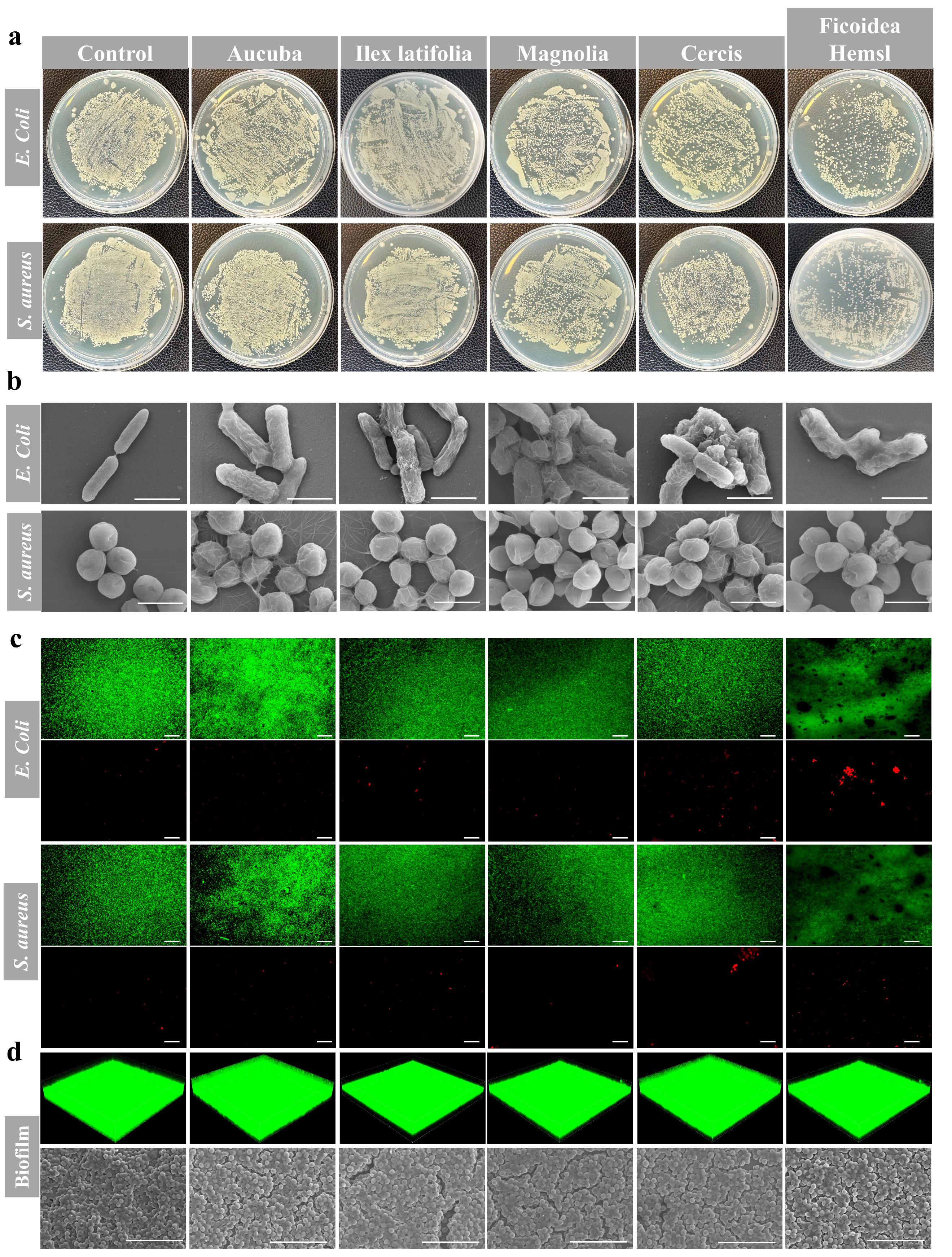


**Fig. S7**. (**a**) Photographic images of the colonies of *E. coli* and *S. aureus* treated by different leaves. (**b**) SEM images of *E. coli* and *S. aureus* treated by leaves (scale bar: 1 μm). (**c**) Living/dead fluorescence images of *E. coli* and *S. aureus* after being treated by different methods (living/dead cells were stained with green/red fluorescence, respectively, scale bar: 20 µm). (**d**) 3D CLSM images (Living cells are stained with green fluorescence, the size of each CLSM image was 1272 µm×1272 µm) and SEM images (scale bar: 10 μm) of *S. aureus* biofilms after being treated by different leaves.

**Supplementary Information Section 8 The content of lignin and polyphenols in leaves and twigs**


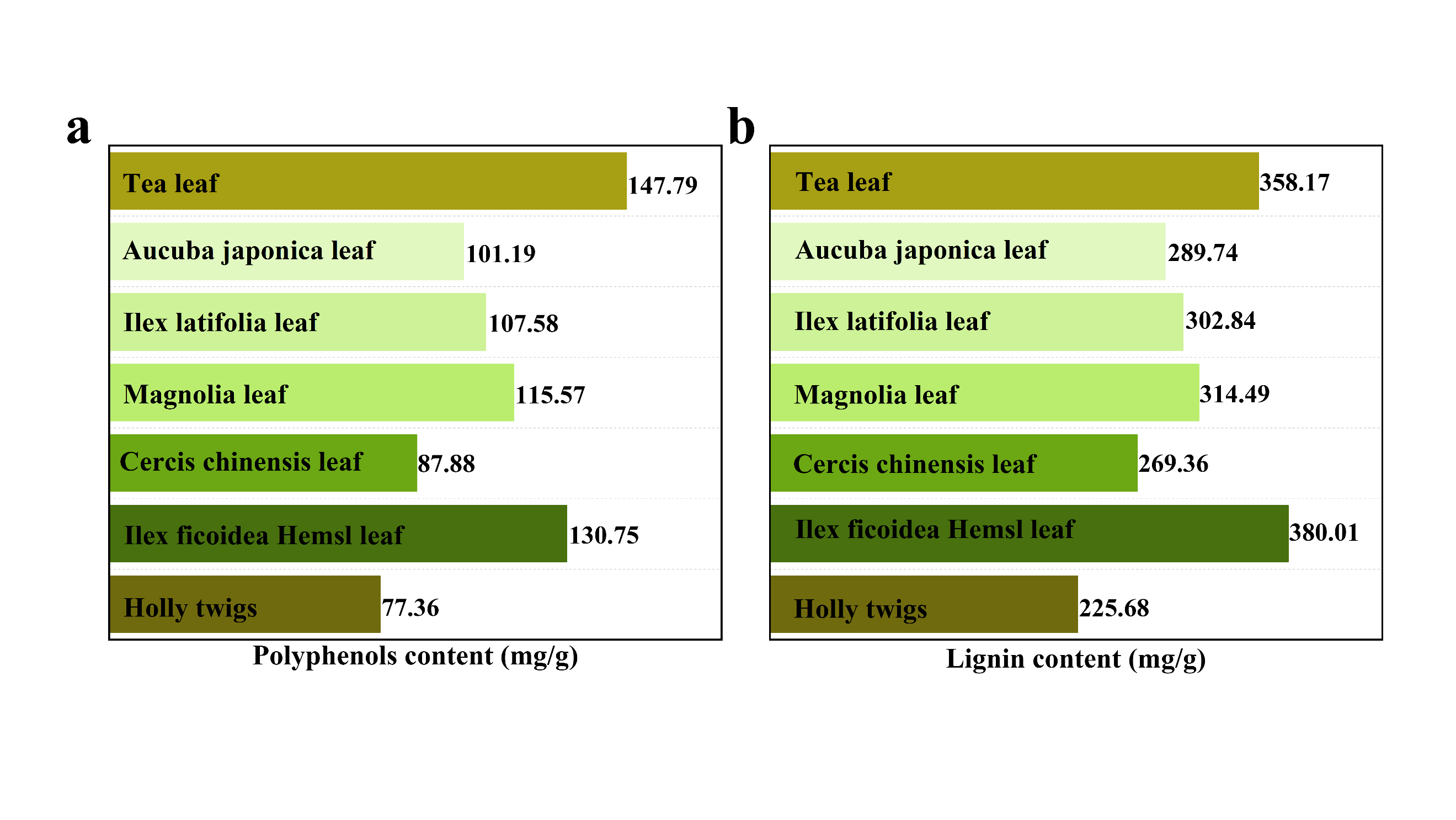


**Fig. S8.** The content of lignin and polyphenols in different samples.

**Supplementary Information Section 9 Antibacterial activities of the Twigs water@Cu.**

**
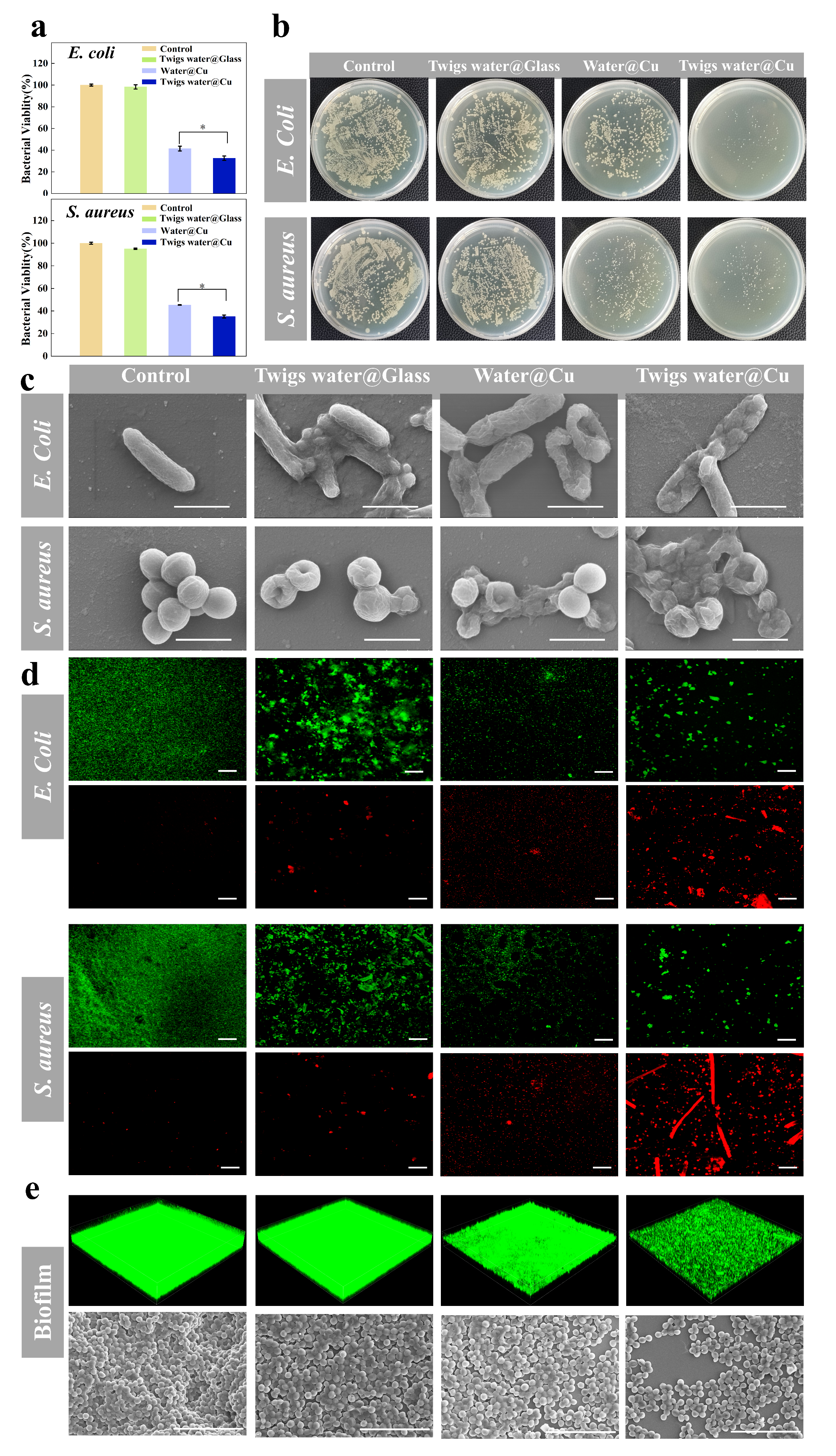
**

**Fig. S9.** (**a**) Bacterial viability of *E. coli* and *S. aureus* treated by Twigs water@Glass, Water@Cu and Twigs water@Cu. (**b**) Photographic images of the colonies of *E. coli* and *S. aureus* treated by different methods. (**c**) SEM images of *E. coli* and *S. aureus* treated by PBS, Twigs water@Glass, Water@Cu and Twigs water@Cu. (scale bar: 1 μm). (**d**) Living/dead fluorescence images of *E. coli* and *S. aureus* after being treated by different methods (living/dead cells were stained with green/red fluorescence, respectively, scale bar: 20 µm). (**e**) 3D CLSM images (Living cells are stained with green fluorescence, the size of each CLSM image was 1272 µm×1272 µm) and SEM images (scale bar: 10 μm) of preformed *E. coli* and *S. aureus* samples for examination of inhibitory potency after treated by PBS, Twigs water@Glass, Water@Cu and Twigs water@Cu.
